# cGAS-like receptors: new RNA sensors in *Drosophila*

**DOI:** 10.1038/s41392-021-00785-z

**Published:** 2021-10-25

**Authors:** Yao Fan, Long Zhang, Fangfang Zhou

**Affiliations:** 1 School of Medicine, Zhejiang University City College, Hangzhou, Zhejiang 310015 China; 2 MOE Laboratory of Biosystems Homeostasis and Protection and Innovation Center for Cell Signaling Network, Life Sciences Institute, Zhejiang University, Hangzhou, 310058 China; 3 Institute of Biology and Medical Sciences, Soochow University, Suzhou, Jiangsu 215123 China

**Keywords:** Innate immunity, Infection

Recently, two back-to-back studies (Slavik et al.^1^ and Holleufer et al.^2^) were published in *Nature*, identifying that cGAS-like receptors (cGLRs) extensively exist in *Drosophila* and act as nucleic acid sensors in the antiviral immune system. Furthermore, they also provided evidence demonstrating that cGLRs in *Drosophila* can sense dsRNA and produce 3′2′-cGAMP (cG[3′–5′]pA[2′–5′]p) as a second messenger that activates the Sting-dependent antiviral immune response.

cGAS has been shown to be a major sensor of dsDNA in mammalian cells. Upon cytosolic DNA binding, cGAS undergoes a conformational change to an active state and catalyzes the synthesis of ATP and GTP into the second-messenger molecule 2ʹ3ʹ- cGAMP (cG[2′–5′]pA[3′–5′]p) which can bind to and activate STING. Subsequently, activation of STING leads to the recruitment and phosphorylation of the transcription factors IRF3 and NF-κB through the kinases TBK1 and IKKε, ultimately triggering the production of type I interferons and proinflammatory cytokines.^3^ The human oligoadenylate synthase (OAS) family of proteins are homologs of cGAS. They can sense cytosolic dsRNA and synthesize 2ʹ–5ʹ‑linked oligoadenylates that act as second messengers to activate RNase L to degrade host and viral RNA. Kranzusch and colleagues previously conducted a systematic biochemical screening for bacterial signaling nucleotides, and they discovered a broad family of cGAS/DncV-like nucleotidyltransferases (CD-NTases), which are structurally conserved families that can utilize both purine and pyrimidine nucleotides to synthesize diverse cyclic dinucleotides (CDNs) and play a key role in bacterial antiviral immune response.^4^ Whether a similar role exists widely in eukaryotes needs to be further clarified.

**Fig. 1:**
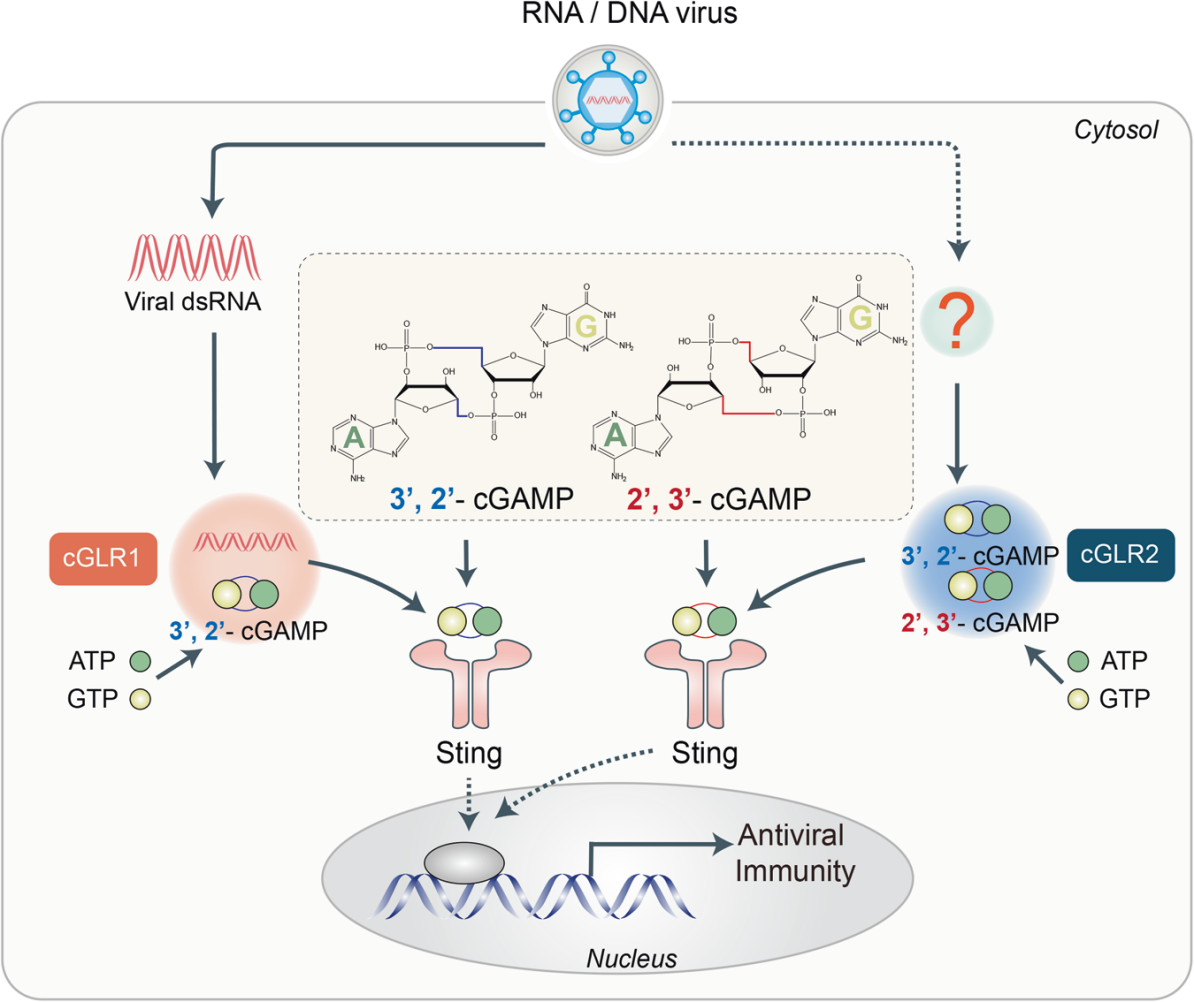
Functions of cGAS-like receptors (cGLRs) in Drosophila immune responses. cGLR1 and cGLR2 are two cGLRs in Drosophila, cGLR1 can response to RNA/DNA virus, while cGLR2 is not definite. The details of DNA virus activating cGLRs are not clear. cGLR1 requires dsRNA to be activated and synthesize 3ʹ2ʹ-cGAMP as a second messenger. The ligand of cGLR2 is unknown, while it can trigger antiviral immunity through synthesizing 3ʹ2ʹ-cGAMP and 2ʹ3ʹ-cGAMP. Sting-dependent pathway is activated after combining the second messengers

Slavik et al.^1^ identified a series of cGLRs in *Diptera* that were similar in structure and enzyme function to cGAS, based on sequence analysis and systematic biochemical screening. However, unlike cGAS, these cGLRs only sensed dsRNA but not dsDNA. They identified the *Drosophila melanogaster* protein CG12970, as a potential cGLR, and was named *Dm*-cGLR1. *Dm*-cGLR1 also functioned as a dsRNA sensor to activate STING-dependent signaling in a mammalian cell ectopic expression model. The mechanism by which dsRNA activated *Dm*-cGLR1 is illustrated. Slavik et al.^1^ purified a nucleotide product synthesized by *Dm*-cGLR1 and found that it had a distinct peak pattern on the C18 chromatography migration profile with the 2ʹ3ʹ-cGAMP synthesized by cGAS, and confirmed that this nucleic acid product was 3'2’-cGAMP (cG[3ʹ–5ʹ]pA[2ʹ–5ʹ]p). Interestingly, *Drosophila* Sting tended to form a stable complex with 3ʹ2’-cGAMP instead of 2'3’-cGAMP in vitro. The in vivo assay also confirmed that Sting preferentially responded to the 3'2ʹ-cGAMP signal. They next determined the structural features of the Sting CDN binding domain in complex with 3′2′-cGAMP, and observed unique ligand-binding pocket adaptations that are widely conserved in *Diptera*. In addition, using an in vivo assay in *D. melanogaster*, Slavik et al.^1^ confirmed that Sting relied on 3′2′-cGAMP to recognize RNA virus infection and activate antiviral immunity. Holleufer et al.^2^ carried out a different research strategy. They identified two homologous proteins of cGAS in *D. melanogaster* through sequence comparison, which contained the conserved active site of CDN synthetase and were named cGLR1 (the same protein as *Dm*-cGLR1 identified by Slavik et al.^1^) and cGLR2. Holleufer et al.^2^ subsequently used the *Drosophila* S2 cell line and transgenic or knockout fly models to confirm that both cGLR1 and cGLR2 can activate the downstream pathways of Sting and present different response patterns. In contrast to the in vitro enzymology experiments of Slavik et al.,^1^ multiple virus infection models verified that cGLR1 could participate in the immunity of RNA and DNA virus infections, while cGLR2 was not definite. An ectopic expression model in human cell lines revealed that activation of cGLR1 required dsRNA, but cGLR2 remained unknown. Furthermore, Holleufer et al.^2^ detected cGLR2 synthesized two second messengers, 3ʹ2ʹ-cGAMP and 2ʹ3ʹ-cGAMP, in approximately equivalent amounts. Moreover, 3′2ʹ-cGAMP seemed to be more effective in activating downstream pathways in insect cells (Fig. 1).

These two groups analyzed the function of cGLRs in *Drosophila* in detail and are the first clarified sensors of nucleic acids that induce antiviral immune responses in insects. These studies are of great significance for the in-depth understanding of insect antiviral immune recognition. In addition, 3′2ʹ-cGAMP was identified as a functional CDN for the first time, which plays an important role as a second messenger in antiviral immunity.

It currently remains unknown how nucleic acids are recognized in *Drosophila*. RNAi and direct cleavage of pathogen RNA have been regarded as the main mechanisms to resist the invasion of pathogens.^1^ It has also been reported that Sting plays a vital role in resistance to RNA virus, but the role of CDNs remains unknown.^5^ Thus, the discovery of cGLRs and 3′2ʹ-cGAMP combined with Sting-NF-κB axis constitutes an intact pathway in response to the stimuli of the virus. There may be a variety of cGLRs in nature, which sense different nucleic acids or other stimuli and synthesize diverse CDNs as second messengers. In different species, the mechanism of cGLR activation may vary, which may provide some insight into the evolution of cGLRs. Future studies may determine whether there is a functional homologous protein of cGAS in human cells. More structural information will help us unravel these mysteries. Also, the identification of 3ʹ2ʹ-cGAMP provides a deeper insight into the small molecule second messenger in cells. Observation of the mass spectrometer data implied that other CDNs may be produced in the process of immune activation and function in some situations.^2^ In addition, whether other second messengers play a role in the process of human immune activation is also a question worth researching.

In brief, research on cGLRs helps to explain how signals of pathogen invasion are detected and transferred in *Drosophila*. This also has strong demonstration for exploration in other species, especially humans. It is obvious that cGLRs may belong to the same family as CD-NTases, according to their definition and similar action mode, which may represent a large class of signal pathway.^4^ They are activated by specific stimuli and then synthesize cyclic nucleotide second messengers to activate specific effectors and initiate signal transduction, whose function may be beyond antiviral immunity.
